# Safety and tolerability of Elvitegravir/Cobicistat/Emtricitabine/Tenofovir Disoproxil fumarate in a real life setting: Data from surveillance cohort long-term toxicity antiretrovirals/antivirals (SCOLTA) project

**DOI:** 10.1371/journal.pone.0179254

**Published:** 2017-06-20

**Authors:** Nicola Squillace, Elena Ricci, Tiziana Quirino, Andrea Gori, Alessandra Bandera, Laura Carenzi, Giuseppe Vittorio De Socio, Giancarlo Orofino, Canio Martinelli, Giordano Madeddu, Stefano Rusconi, Paolo Maggi, Benedetto Maurizio Celesia, Laura Cordier, Francesca Vichi, Leonardo Calza, Katia Falasca, Antonio Di Biagio, Giovanni Francesco Pellicanò, Paolo Bonfanti, for the CISAI Study Group

**Affiliations:** 1 Infectious Diseases Clinic, Azienda Socio Sanitaria Territoriale di MONZA, San Gerardo Hospital-University of Milano-Bicocca, Monza, Italy; 2 Department of Infectious Diseases, Azienda Socio Sanitaria Territoriale Fatebenefratelli Sacco, Milano, Italy; 3 Unit of Infectious Diseases, Azienda Socio Sanitaria Territoriale della Valle Olona–Busto Arsizio (VA), Italy; 4 Unit of Infectious Diseases, Santa Maria Hospital, Perugia, Italy; 5 Unit of Infectious Diseases, Amedeo di Savoia Hospital, Torino, Italy; 6 Unit of Infectious Diseases, Careggi Hospital, Firenze, Italy; 7 Department of Clinical and Experimental Medicine, University of Sassari, Sassari, Italy; 8 Infectious Diseases, Department of Biomedical and Clinical Science Luigi Sacco, University of Milan, Milan, Italy; 9 Infectious Disease Clinic, University of Bari, Italy; 10 Unit of Infectious Diseases, Garibaldi Hospital, Catania, Italy; 11 Unit of Infectious Diseases, Santa Maria Annunziata Hospital, Firenze, Italy; 12 Department of Infectious Diseases, S.Orsola Malpighi Hospital, Bologna, Italy; 13 Clinic of Infectious Diseases, Department of Medicine and Science of Aging, University “G. d’Annunzio” Chieti-Pescara, Chieti, Italy; 14 Infectious Diseases, San Martino Hospital Genoa, University of Genoa, Genoa, Italy; 15 Infectious Diseases, G. Martino Hospital -University of Messina, Messina, Italy; 16 Unit of Infectious Diseases, A. Manzoni Hospital, Lecco, Italy; University of Ottawa, CANADA

## Abstract

**Objectives:**

The study aim was to evaluate the impact on Liver and Kidney toxicity of the single tablet regimen Elvitegravir/Cobicistat/Emtricitabine/Tenofovir Disoproxil Fumarate **(**EVG/COBI/FTC/TDF) on Antiretroviral Therapy (ART) experienced or naïve patients.

**Methods:**

Patients initiating EVG/COBI/FTC/TDF were enrolled in the SCOLTA project, a multicenter observational study reporting grade 3–4 Adverse Events in subjects beginning new antiretroviral drug regimens. In this analysis, patients were evaluated at T0 (baseline), T1 (six months) and at T2 (twelve months).

**Results:**

A total of 329 patients were enrolled, and 280 (85.1%) of these had at least one follow-up visit. Median observation time was 11 months (IQR 7.0–15.5). Two hundred and two patients (72.1%) were ART experienced and 78 (27.9%) ART naive. Prevalence of HCV-co-infection was 21.4%. At T1, we observed a significant decline in estimated glomerular filtration rate (eGFR), both in experienced and naive patients (mean change from T0–7.5 ± 12.8 ml/min, -15.5 ± 17.8 ml/min, respectively, p = 0.0005), which was confirmed at T2 (mean change from T0–8.2 ± 15.8 ml/min, -17.6 ± 19.4 ml/min, respectively, p = 0.001). Regarding aspartate aminotransferase (AST) and alanine transaminase (ALT) grade 1–2 modifications, no significant differences were observed between experienced and naïve subjects, but an increased prevalence of abnormal liver function test was observed in patients with chronic HCV infection (p<0.001).

**Conclusions:**

A significant decline in eGFR was observed in patients initiating EVG/COBI/FTC/TDF in the first 6 months, with no significant worsening occurring at 12 months vs. 6 months of therapy. Patients with chronic HCV infection were at higher risk to develop abnormal liver tests.

## Introduction

Most studies evaluating the safety of Elvitegravir/Cobicistat/Emtricitabine/Tenofovir-Disoproxil fumarate (EVG/COBI/FTC/TDF) have described a significant reduction in estimated glomerular filtration rate (eGFR) during the first four weeks of treatment, and few changes through 48 weeks, both in naive and experienced patients [1–5]. In two studies performed on naive patients, median eGFR change at 48 weeks of treatment was -12.7 and -14.3 ml/min [1, 2], while a median creatinine increase of 0.06 and 0.12 mg/dl was observed in experienced subjects at 48 weeks of treatment [3, 4]. In a study evaluating naive patients with mild to moderate renal impairment, median eGFR change from baseline was -7.6 ml/min at week 48 [5].

Elevations in alanine transaminase (ALT) and aspartate aminotransferase (AST) were observed less frequently during EVG/COBI/FTC/TDF treatment, compared to Tenofovir/emtricitabine/efavirenz (TDF/FTC/EFV) and tenofovir/emtricitabine/atazanavir/ritonavir (TDF/FTC/ATV/r) regimens. Such events occurred in 15–17% of naive patients [1, 2] and 2% of experienced ones [4] on EVG/COBI/FTC/TDF, vs. 31–34% of naive patients on TDF/FTC/EFV [1], in 22% of those on TDF/FTC/ATV/r as their first line treatment [2] and in 1% of experienced patients on Protease Inhibitor regimens [4].

Few patients in these studies were co-infected with HBV and HCV, and a very small proportion of subjects had CD4 +T cell counts <200 cell/micrL [1–4]. The most frequent adverse events (AE) during the first 48 weeks of treatment with EVG/COBI/FTC/TDF were: diarrhea (7–23%), nausea (7–21%), upper respiratory tract infections (8–15%) and headache (6–15%) [1–4]. Insomnia and depression were reported in about 3–9% of patients[1–4] and skin rash was experienced only by 0.3–6.0% of patients [1, 2].

In Italy, the number of patients with HIV-HCV co-infection has increased to 45% [6] and most patients start therapy with advanced chronic HCV infection (Hepatic fibrosis F3/F4), due to Italian Pharmaceutical Agency restrictive rules for treatment of HCV infection [7]. Our primary aim was to evaluate the impact of this regimen on liver and kidney function in both naïve and experienced patients receiving combination ART (cART). Our secondary purpose was to identify the overall frequency of adverse drug reactions.

## Materials and methods

Patients initiating EVG/COBI/FTC/TDF were enrolled in the Surveillance Cohort Long-term Toxicity Antiretrovirals/antivirals (SCOLTA) project, a prospective, multicenter observational study created to assess the incidence of AE in patients receiving new antiretroviral drugs in clinical practice. The project has an internet site (http://www.cisai.info) where grade III and IV AE are recorded, according to the Division of AIDS Tables [8]. The SCOLTA Project currently includes three cohorts: dolutegravir, EVG/COBI/FTC/TDF and darunavir/cobicistat. Patients undergo follow-up at 6-month intervals and AEs are notified when they are clinically observed. Details are described elsewhere [9]. The study protocol was approved by the local ethics committee of the coordinating center at Hospital “L. Sacco”-University of Milan on 18 September 2002. A new protocol amendment was submitted and approved on 13 June 2013 by the same ethics committee. This last version was approved by local ethics committee of each group and written consent was obtained from all participants.

Patients were evaluated at T0 (baseline), T1 (six months) and T2 (twelve months). eGFR was calculated as mL/min/1.73 m^2,^ using the Modification of Diet in Renal Disease (MDRD) formula: eGFR = 186 * serum creatinine (mg/mL)^-1.157^ * age (years)^-0.203^ * 1.210 (if black) * 0.742 (if female).

As far as transaminase increase, grade 1–2 events were defined as 1.25-<5.00 x upper normal level (AST: 40 mg/dL; ALT 35 mg/dL) if baseline values were normal, and 1.25-<5.00 x baseline value if an abnormal value was present at baseline. Grade 3–4 were recorded in the online system.

HCV infection was defined as the presence of HCV-Antibodies (Ab); HCV chronic infection as the presence of HCV-Ab and HCV-RNA.

Categorical and discrete variables were described as frequency and percentage (%). Continuous variables were described using mean and standard deviation (SD) if normally distributed, and median and interquartile range (IQR) if not normally distributed. At univariate analysis, groups were compared using chi-square for categorical variables (Person chi-square or Fisher exact test or Mantel-Haenszel test as appropriate) and analysis of variance for continuous variables, or via non-parametric tests for not normally distributed continuous variables. Repeated measures were analyzed as change from baseline. A general linear model was used to include potential confounders in the multivariate analysis.

Hazard ratios (HR) and 95% confidence intervals (CI) were calculated for treatment interruption, and logistic regression was used to adjust simultaneously for the potentially confounding effects of selected variables, according to the Cox model. Variables with p<0.20 at univariate were subsequently included in the model equation as appropriate (i.e., scores were adjusted only for variables not included in their calculation and correlated variables were included in turn). Statistical analysis was performed using the SAS/STAT statistical package (version 9.4; SAS Institute Inc., Cary, North Carolina, USA).

## Results

Between January 2014 and December 2016, 329 patients were enrolled, 280 (85.1%) of which had at least one follow-up visit. Two hundred and two (72.1%) were ART experienced and 78 (27.9%) were treatment naïve. Prior to the cohort drug, ART experienced patients were on Protease Inhibitors (115, 56.9%) or Non-Nucleoside Reverse Transcriptase Inhibitors (51, 25.3%) or Integrase Strand Transfer Inhibitors (35, 17.3%)-based treatment: 145 (71.8%) were virologically suppressed when starting EVG/COBI/FTC/TDF and 161 (79.7%) were already on a regimen including TDF.

HCV-Antibody (Ab) positive patients were 61 (21.8%, 59 experienced and two naive); The baseline characteristics of the sample are shown in Table 1.

**Table 1:** Patient’s characteristics (n = 280.

Variables		Experienced	Naive	P
		N = 202 (72.1%)	N = 78 (27.9%)	
**Males**		147 (72.8)	65 (83.3)	0.06
**Risk factor IVDU**		50 (24.8)	2 (2.6)	<0.0001
**CDC Stage C**		52 (25.7)	14 (18.0)	0.05
**Detectable HIV-RNA**		57 (28.2)	78 (100)	<0.0001
**HCV-Ab positivity¶**		59 (30.1)	2 (2.7)	<0.0001
	Detectable HCV-RNA	30	1	
	Undetectable HCV-RNA	19	0	
	Missing HCV-RNA	10	1	
**eGFR BL^**				0.02*
	>90	101 (50.5)	50 (70.1)	
	80–89	44 (22.0)	14 (18.2)	
	70–79	35 (17.5)	6 (7.8)	
	<70	20 (10.0)	3 (3.9)	
		Mean (SD) or median (IQR)	Mean (SD) or median (IQR)	
**Age (years)**		45.7 (9.5)	39.2 (11.9)	<0.0001
**BMI (Kg/m**^**2**^**)**		24.1 (3.4)	22.9 (2.7)	0.0009
**CD4+T cells (cells/ml)**		578 (356)	377 (280)	<0.0001
**HIV-RNA (cp/mm**^**3**^**)**		24 (19–67)	50,042 (8,040–117,171)	<0.0001
**cART duration (years)**		8.0 (3.3–16.8)	0	
**Total cholesterol (mg/dL)**		191.1 (44.9)	158.7 (37.5)	<0.0001
**HDL cholesterol (mg/dL)**		43.2 (12.6)	40.1 (13.6)	0.09
**Triglycerides (mg/dL)**		140 (91–196)	92 (70–121)	<0.0001
**Blood glucose (mg/dL)**		94.3 (29.1)	89.8 (17.1)	0.21
**AST (IU/L)**		24 (20–35)	24 (20–32)	0.88
**ALT (IU/L)**		28 (18–46)	27 (17–38)	0.27
**Creatinine (mg/dL)**		0.90 (0.18)	0.86 (0.16)	0.047
**Alcaline phosphatase (IU/L)**		118.9 (76.4)	88.2 (42.0)	0.005
**Fosforemia (mg/dL)**		3.19 (0.68)	3.10 (0.68)	0.46

* chi-square for trend

¶ 10 missing

^ 3 missing

IVDU intravenous drug users, CDC center for disease control, HCV hepatitis C virus, Ab antibody, eGFR estimated glomerular filtration rate, BL baseline, SD standard deviation, IQR interquartile range, BMI body mass index, cART antiretroviral therapy, HDL high density lipoprotein, AST aspartate aminotransferase, ALT alanine transaminase.

Over time, CD4+ T cells change from baseline was 29 (±188) at T1 and 32 (±180) at T2 in experienced patients, and 125 (±167) at T1 and 190 (±162) at T2 in naïve ones (p<0.0001). Among those who had reached the observation visit, HIV-RNA was still detectable in 18.4% of experienced and 23.0% of naïve subjects at T1 (p = 0.40), and respectively in 13.7% and 14.6% at T2 (p = 0.88).

### Kidney and liver adverse events

**Fig 1:**
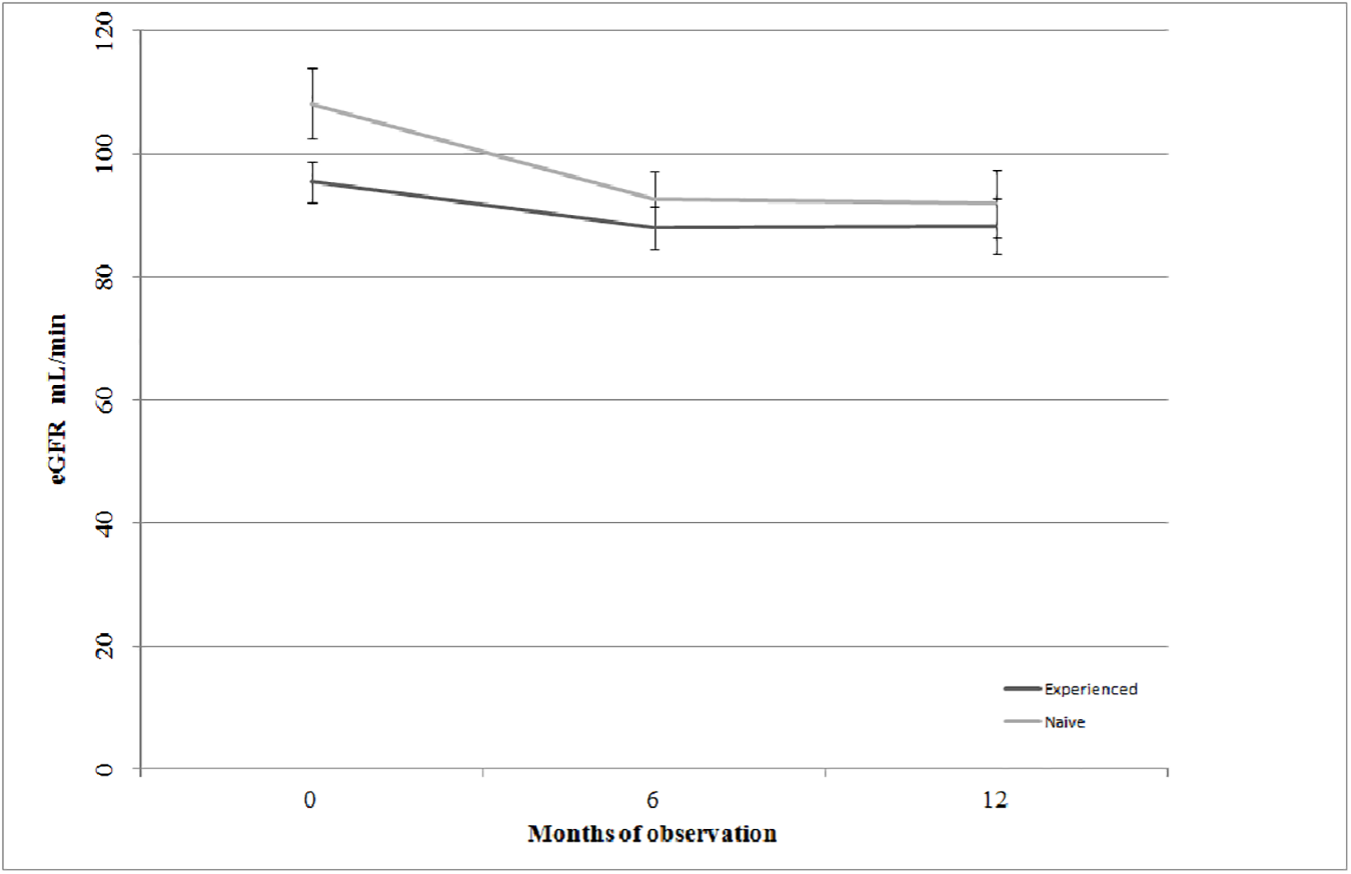
eGFR change from baseline: Means and 95% confidence intervals”. eGFR = estimated Glomerular Filtration Rate.

At baseline, the mean eGFRs were significantly different between experienced and naïve patients. However, they tended to be similar at follow-up. Consistently, changes from baseline were significantly higher in naïve than in experienced group (see Table 2 and Fig 1).

**Table 2:** Adverse events.

Variables			Experienced	Naive	P
			N = 202 (72.1%)	N = 78 (27.9%)	
**eGFR**					
	T0		95.4 (24.3)	108.2 (26.0)	0.0001
	T1		87.9 (24.8)	92.6 (20.4)	0.13
	T2		88.2 (27.3)	91.9 (19.9)	0.39
	T1-T0		-7.5 (12.8)	-15.5 (17.8)	<0.0001
	T2-T0		-8.2 (15.8)	-17.6 (19.4)	0.001
	T2-T1		0.6 (15.6)	-1.2 (12.2)	0.46
**eGFR <70 at T1 or T2**					
	with eGFR ≥70 at BL (n = 178 exp./74 naive)		34 (19.1)	10 (13.5)	0.29
	with eGFR<70 at BL (n = 20 exp./3 naive)		19 (95.0)	3 (100.0)	1.00
eGFR category as compared to BL*					
	Worse		88 (43.6)	34 (43.6)	0.60
	Same		92 (45.5)	39 (50.0)	
	Better		22 (10.9)	5 (6.4)	
**Liver enzyme increase**					
	Grade 1–2		17 (8.5)	3 (3.8)	0.40
	Grade 3–4 or causing treatment interruption		2 (1.0)	1 (1.3)	
**Grade 3–4 adverse events not leading to treatment interruption**			12 (5.9)	1 (1.3)	0.01
	Central nervous system		3	0	
	Cardiovascular disease		2	0	
	Diarrhea		1	0	
	Blood lipid increase		2	0	
	Muscle pain		1	0	
	Other#		3	1	
**Treatment interruptions**			46 (22.8)	8 (10.3)	0.01
**Reasons for treatment interruptions**					
	Virological failure		10 (21.7)	1 (12.5)	0.25
	Death		2 (4.3)	0	
		Hepatic cancer	1	0	
		Overdose	1	0	
	Adverse events		11 (23.9)	5 (62.5)	
		Gastrointestinal	4	1	
		Kidney related	2	2	
		Liver related	1	1	
		Skin	1	0	
		Central nervous system	1	0	
		Other§	2	1	
	Poor adherence/patient’s choice		9 (19.6)	2 (25.0)	
	Other: starting DAA treatment		4 (8.7)	0	
	Lost to follow-up		10 (21.7)	0	

eGFR estimated glomerular filtration rate, BL baseline, DAA direct antiviral agents

*At least +1/-1 move through categories <70, 70–79, 80–89,≥90 mL/min

#Karposi sarcoma, acute Hepatitis C infection, pneumonia, fever

§ Multiple skin infections, erectile dysfunction, intolerance

After adjusting for HCV-RNA positivity, CDC stage, body mass index (BMI), CD4+ T cells and eGFR at T0, change from baseline was statistically significant both in experienced and naive subjects, at T1 and T2 versus T0. No marked difference was observed between adjusted and unadjusted means: -6.0 (standard error (SE) 1.6) and -6.4 (SE 2.1) for experienced subjects at T1 and T2 respectively, and -11.5 (SE 2.6) and -15.2 (SE 4.1) for naive patients.

Changes from T1 to T2 were negligible: 0.6 ± 15.6 for experienced and -1.2 ± 12.2 ml/min for naive; adjusted means were 1.0 (SE 2.0) and -3.4 (SE 3.8), showing no statistically significant difference from zero.

Out of 178 experienced patients with at least one follow-up visit and eGFR at T0 ≥70 mL/min, 34 (19.1%) experienced a decline of eGFR <70 mL/min at T1 and T2; the corresponding figure for 74 naive subjects was ten (13.5%), a proportion not significantly different from that of experienced patients (P = 0.29).

Stratifying for eGFR value at baseline (<70, 70–79, 80–89, and ≥90 mL/min), a similar proportion of experienced and naive patients showed a worsening or an improvement of renal impairment stage (p = 0.60). However, renal impairment was cause for interruption only in four patients, two experienced and two naïve.

Regarding liver AE, grade 3–4 were recorded in the designated form and grade 1–2 were calculated using follow-up laboratory data. An experienced patient who was HCV-Ab negative at baseline and presented acute HCV infection during follow-up was excluded from the analysis of liver-related AEs.

No significant differences between experienced and naive patients were observed during follow-up (Table 2) except for treatment interruption that were more frequent in experienced patients. However, after excluding patients who interrupted because of interactions with directly antiviral agents (DAAs) no significant differences were confirmed. As for HCV status, 11 liver AEs occurred among 208 HCV-Ab negative (5.3%), one among 19 HCV-Ab positive/HCV-RNA negative (5.3%), eight among 31 HCV-RNA positive (25.8%), two among 11 HCV-Ab positive subjects with missing HCV-RNA (18.2%), and one among 10 patients with unknown HCV-RNA status (10%). When including, at the same time, HCV-Ab and HCV-RNA status and treatment status (naïve/experienced) in the analysis, no statistically significant difference was found between naïve and experienced patients (p = 0.40), whereas HCV-RNA positive subjects had a significantly higher proportion of liver related AEs (Mantel-Haenszel chi-square p = 0.001, excluding missing HCV-RNA patients).

### Safety-adverse events and treatment interruptions

During the observation period, 54 (19.3%) patients withdrew treatment: 11 were virological failures, four switched to other treatments because of DAAs initiation, ten were lost to follow-up, and eleven chose to interrupt. The 11 patients with virological failure were mostly experienced patients (ten vs. one), with a high prevalence of HCV-Ab positivity (45%); median time to interruption was 13 months (range 1–19); their mean CD4+T cells change from baseline was -21 (±79) and -8 (±251) at T1 and T2 respectively. One patient died from hepatic cancer and one from accidental drug overdose. Overall, 16 patients (5.7%) interrupted their treatment because of AEs (ten grade 1–2, six grade 3–4).

Four patients interrupted for kidney-related events (impaired creatinine clearance); five because of gastrointestinal events; two had liver-related events (one liver decompensation in an experienced HCV-co-infected patient and one transaminase increase in a naive HCV-Ab negative subject). Other reasons for interruption were: erectile dysfunction, skin rash, insomnia, intolerance.

Excluding four patients who withdrew from treatment because they had started DAAs, interruptions were more frequent among experienced than naive patients, but not significantly (HR 1.58, 95% CI 0.73–3.39, p = 0.24): after adjustment for potential confounders measured at study entry (gender, age, BMI, CD4+T cells, CDC stage, HCV-RNA status), the risk showed a slight increase (HR 2.14, 95% CI 0.92–5.00, -p = 0.08). The only one significant predictor of treatment interruption was CD4+ T cells at study entry (HR 0.92, 95%; 0.86–0.97 by 50 additional cells/mL, p = 0.003). Models including CD4+T cells at T1 and T2 provided similar results.

As far as grade 3–4 AEs not leading to treatment interruption, they occurred mainly in experienced patients (12/13, 92.3%): three central nervous system (CNS) events (headache, anxiety, sleepwalk) were considered to have possibly been related to EVG/COBI/FTC/TDF. Overall, grade 3–4 AEs occurred in 19 subjects: after adjustment for potential confounders as measured at study entry (gender, age, BMI, CD4+T cells, CDC stage, HCV-RNA status) HR for first grade 3–4 AE in experienced patients was 1.96 (95% CI 0.38–10.14, p = 0.42), with naive patients used as the reference category.

## Discussion

In this study, we took a snapshot of EVG/COBI/FTC/TDF use in a real life Italian setting.

Observed rates of discontinuation from significant reduction of creatinine clearance in experienced patients were similar to rates seen in RCTs [3, 4], while such interruptions were of a comparatively higher proportion in our naive patients, with a percentage of 2.6% vs. 0.3–1% [1, 2]. This result may be due to the relatively small sample of naïve subjects in our study (78 patients), since these patients had significantly better eGFR profiles at baseline than the experienced group. However, the 95% confidence interval for this proportion was wide (0.7–8.8%), and included the estimates observed in other studies [1, 2].

One reason for the general significant eGFR reductions which we observed could be that a small group of patients initiated EVG/COBI/FTC/TDF with an eGFR<70ml/min, despite contra-indication. Interestingly, we observed that, in clinical practice, eGFR calculation is often not carried out, possibly because physicians are used to suppose that normal creatinine levels are an indication of normal eGFR levels when, in fact, levels of the former can be normal, even in presence of eGFR lower than 70 ml/min (i.e. in older patient). However, among 23 patients who had eGFR< 70 mL/min at enrollment, only three experienced subjects withdrew because of AEs: one because of impaired creatinine clearance and two for other events.

Finally, a significant eGFR reduction of in both naive and experienced patients was observed. This was not a surprise, given the known inhibition of the multi drug and toxin extrusion-1(MATE-1) renal transporter, which is involved in tubular secretion of creatinine [10]. We confirmed the data of RCTs on the stabilization of these effects after the first 4 weeks of treatment.

We observed a more marked eGFR reduction in naive patients, likely because they had never been exposed to ritonavir, which itself affects tubular secretion of creatinine and can partially inhibit MATE-1 [11] and to tenofovir that, combined with COBI as first line regimen, might have been the cause of this decline.

Contrastingly, we observed significantly lower eGFR in experienced than in naive patients, even though the decrease in eGFR was more marked in naïve ones. Considering the whole sample, during the follow-up period, the proportion of patients with eGFR<70 increased by 2 to 3 fold. This occurrence was not considered to be sufficient reason for interruption, probably because COBI documented effect on creatinine secretion invalidates the use of creatinine in calculating eGFR. Recently, Post et al. published a study about kidney safety of EVG/COBI/FTC/TDF in patients with eGFR≥50 ml/min [5]: they calculated eGFR using cystatin C, which is not influenced by COBI, and observed safety levels similar to those reported in studies conducted on patients with eGFR≥70 ml/min. It is probable that in our study, physicians’ decisions to continue EVG/COBI/FTC/TDF were influenced by such considerations.

Rates of treatment interruption due to both gastrointestinal and liver events were similar in the experienced group as well as in naive patients, as compared to RCTs. However, it should be noted that we observed a very low incidence of gastrointestinal events of any grade. These results could be partially due to a lack of reporting, especially in experienced patients, which represent the majority of our sample. Having experienced a far greater incidence of gastrointestinal events with previous Protease Inhibitors or Non-Nucleoside Reverse Transcriptase Inhibitors based treatments, patient-reported symptoms, both in STRATEGY-PI and in STRATEGY-NNRTI, were significantly reduced [12, 13]. This amelioration of gastrointestinal symptoms has probably contributed in reducing the reports of such events by patients in our cohort.

Although our cohort included a large percentage of patients with HCV-Ab positivity (21.8% of subjects with at least 1 follow-up visit), we did not observe a significant increase in ALT and AST during the first 48 weeks of treatment considering only HCV infection (both HCV-RNA negative and positive). However, fifty percent of patients with grade 3–4 AEs suffered from chronic hepatitis C and a significant higher proportion of patients with positive HCV-RNA experienced liver AE. We confirmed a low incidence of liver events in patients initiating EVG/COBI/FTC/TDF a described in RCTs [1–4] and the higher rates of abnormal AST and ALT and liver decompensation in antiretroviral treated patients with HIV-HCV coinfection as described in literature [14, 15]. Data are lacking about specific hepatic toxicity of EVG/COBI in patient with chronic HCV viral hepatitis. Recent data about use of COBI/EVG on 72 patients with HIV-HBV coinfection showed a normalization of ALT in 50% of patients with abnormal ALT at baseline[16] but patients were mostly with a suppressed HBV-DNA. We could argue that uncontrolled HCV viral replication could be a risk factor for abnormal liver test in patients initiating EVG/COBI/FTC/TDF.

Our study has some limitations. A main limitation is the comparatively small sample size and the subsequent low power, that prevented us from drawing significant conclusions in case of infrequent events. Another limit was the use of creatinine as marker of eGFR that is not so accurate in patients on COBI, we took into account this limitation and compared our data only with studies that used only creatinine like ours. We have no sure data on liver fibrosis stage in patients with HIV-HCV coinfection even if we can suppose that most patients should have a liver stiffness <10 kPa because no one has been treated with direct antiviral agents during the study period since Italian Health System rules provided treatment for HCV only for patients with a liver stiffness ≥ 10 kPa. [7]

## Conclusions

A significant decline in eGFR was observed in patients initiating EVG/COBI/FTC/TDF at 6 month-follow-up, with no significant worsening at 12 months compared to 6 months. Our data suggests the importance of careful monitoring of renal function, both in naïve and in experienced patients who are initiating EVG/COBI/FTC/TDF, especially during the first 4 weeks, even in patients with normal eGFR at baseline. Patients with positive HCV-RNA were at higher risk of developing AST and ALT elevation and liver-related events.

## Supporting information

S1 File Patients’characteristics and adverse events. BMI, body mass index; PI, protease inhibitor; NNRTI, non-nucleoside reverse transciptase inhibitor; INSTI, integrase strand transfer inhibitor; TDF, tenofovir disoproxil fumarate; HCV, hepatitis C virus; Ab, antibody; eGFR, estimated glomerular filtration rate; BL, baseline; SD standard deviation; IQR, interquartile range; cART, antiretroviral therapy; HDL, high density lipoprotein; AST, aspartate aminotransferase; ALT, alanine transaminase; det, detectable; AE, adverse event; GI, gastro-intenstinal, DAA, direct antiviral agents.
(XLSX)

## References

[1] Sax PE, DeJesus E, Mills A, Zolopa A, Cohen C, Wohl D, et al Co-formulated elvitegravir, cobicistat, emtricitabine, and tenofovir versus co-formulated efavirenz, emtricitabine, and tenofovir for initial treatment of HIV-1 infection: a randomised, double-blind, phase 3 trial, analysis of results after 48 weeks. Lancet. 2012;379(9835):2439–48. doi: 10.1016/S0140-6736(12)60917-9 . 22748591 10.1016/S0140-6736(12)60917-9

[2] DeJesus E, Rockstroh JK, Henry K, Molina JM, Gathe J, Ramanathan S, et al Co-formulated elvitegravir, cobicistat, emtricitabine, and tenofovir disoproxil fumarate versus ritonavir-boosted atazanavir plus co-formulated emtricitabine and tenofovir disoproxil fumarate for initial treatment of HIV-1 infection: a randomised, double-blind, phase 3, non-inferiority trial. Lancet. 2012;379(9835):2429–38. doi: 10.1016/S0140-6736(12)60918-0 . 22748590 10.1016/S0140-6736(12)60918-0

[3] Pozniak A, Markowitz M, Mills A, Stellbrink HJ, Antela A, Domingo P, et al Switching to coformulated elvitegravir, cobicistat, emtricitabine, and tenofovir versus continuation of non-nucleoside reverse transcriptase inhibitor with emtricitabine and tenofovir in virologically suppressed adults with HIV (STRATEGY-NNRTI): 48 week results of a randomised, open-label, phase 3b non-inferiority trial. Lancet Infect Dis. 2014;14(7):590–9. doi: 10.1016/S1473-3099(14)70796-0 . 24908550 10.1016/S1473-3099(14)70796-0

[4] Arribas JR, Pialoux G, Gathe J, Di Perri G, Reynes J, Tebas P, et al Simplification to coformulated elvitegravir, cobicistat, emtricitabine, and tenofovir versus continuation of ritonavir-boosted protease inhibitor with emtricitabine and tenofovir in adults with virologically suppressed HIV (STRATEGY-PI): 48 week results of a randomised, open-label, phase 3b, non-inferiority trial. Lancet Infect Dis. 2014;14(7):581–9. doi: 10.1016/S1473-3099(14)70782-0 . 24908551 10.1016/S1473-3099(14)70782-0

[5] Post FA, Winston J, Andrade-Villanueva JF, Fisher M, Liu Y, Beraud C, et al Elvitegravir/cobicistat/emtricitabine/tenofovir DF in HIV-infected patients with mild-to-moderate renal impairment. J Acquir Immune Defic Syndr. 2015;68(3):310–3. doi: 10.1097/QAI.0000000000000476 . 25469527 10.1097/QAI.0000000000000476

[6] De Luca A, Bugarini R, Lepri AC, Puoti M, Girardi E, Antinori A, et al Coinfection with hepatitis viruses and outcome of initial antiretroviral regimens in previously naive HIV-infected subjects. Arch Intern Med. 2002;162(18):2125–32. . 12374521 10.1001/archinte.162.18.2125

[7] Marcellusi A, Viti R, Damele F, Camma C, Taliani G, Mennini FS. Early Treatment in HCV: Is it a Cost-Utility Option from the Italian Perspective? Clin Drug Investig. 2016;36(8):661–72. doi: 10.1007/s40261-016-0414-y . 27234943 10.1007/s40261-016-0414-y

[8] Division of AIDS (DAIDS) Table for Grading the Severity of Adult and Pediatric Adverse Events. https://rsc.tech-res.com/docs/default-source/safety/daids_ae_grading_table_v2_nov2014.pdf?sfvrsn=8 [updated 2014; cited 2017 19 April 2017].

[9] Menzaghi B, Ricci E, Carenzi L, Parruti G, Orofino G, Guastavigna M, et al Safety and durability in a cohort of HIV-1 positive patients treated with once and twice daily darunavir-based therapy (SCOLTA Project). Biomed Pharmacother. 2013;67(4):293–8. doi: 10.1016/j.biopha.2012.12.005 . 23433852 10.1016/j.biopha.2012.12.005

[10] Maggi P, Montinaro V, Mussini C, Di Biagio A, Bellagamba R, Bonfanti P, et al Novel antiretroviral drugs and renal function monitoring of HIV patients. AIDS Rev. 2014;16(3):144–51. . 25102336

[11] Gutierrez F, Fulladosa X, Barril G, Domingo P. Renal tubular transporter-mediated interactions of HIV drugs: implications for patient management. AIDS Rev. 2014;16(4):199–212. . 25350530

[12] Mills A, Garner W, Pozniak A, Berenguer J, Speck RM, Bender R, et al Patient-Reported Symptoms Over 48 Weeks in a Randomized, Open-Label, Phase IIIb Non-Inferiority Trial of Adults with HIV Switching to Co-Formulated Elvitegravir, Cobicistat, Emtricitabine, and Tenofovir DF versus Continuation of Non-Nucleoside Reverse Transcriptase Inhibitor with Emtricitabine and Tenofovir DF. Patient. 2015;8(4):359–71. doi: 10.1007/s40271-015-0129-9 ; PubMed Central PMCID: PMCPMC4529476. 26045359 10.1007/s40271-015-0129-9 PMC4529476

[13] Gathe J, Arribas JR, Van Lunzen J, Garner W, Speck RM, Bender R, et al Patient-Reported Symptoms over 48 Weeks in a Randomized, Open-Label, Phase 3b Non-inferiority Trial of Adults with HIV Switching to Coformulated Elvitegravir, Cobicistat, Emtricitabine, and Tenofovir DF Versus Continuation of Ritonavir-Boosted Protease Inhibitor with Emtricitabine and Tenofovir DF. Patient. 2015;8(5):445–54. doi: 10.1007/s40271-015-0137-9 ; PubMed Central PMCID: PMCPMC4575373. 26286337 10.1007/s40271-015-0137-9 PMC4575373

[14] Lo Re V 3rd, Kallan MJ, Tate JP, Localio AR, Lim JK, Goetz MB, et al Hepatic decompensation in antiretroviral-treated patients co-infected with HIV and hepatitis C virus compared with hepatitis C virus-monoinfected patients: a cohort study. Ann Intern Med. 2014;160(6):369–79. doi: 10.7326/M13-1829 ; PubMed Central PMCID: PMCPMC4254786. 24723077 10.7326/M13-1829 PMC4254786

[15] Mena A, Meijide H, Rodriguez-Osorio I, Castro A, Poveda E. Liver-related mortality and hospitalizations attributable to chronic hepatitis C virus coinfection in persons living with HIV. HIV Med. 2017 doi: 10.1111/hiv.12502 . 28230318 10.1111/hiv.12502

[16] Gallant J, Brunetta J, Crofoot G, Benson P, Mills A, Brinson C, et al Brief Report: Efficacy and Safety of Switching to a Single-Tablet Regimen of Elvitegravir/Cobicistat/Emtricitabine/Tenofovir Alafenamide in HIV-1/Hepatitis B-Coinfected Adults. J Acquir Immune Defic Syndr. 2016;73(3):294–8. doi: 10.1097/QAI.0000000000001069 ; PubMed Central PMCID: PMCPMC5172523. https://rsc.tech-res.com/docs/default-source/safety/daids_ae_grading_table_v2_nov2014.pdf?sfvrsn=8 27171740 10.1097/QAI.0000000000001069 PMC5172523

